# The Treatment of Wounds Infected with Bacillus Pyocyaneus

**Published:** 1917-11

**Authors:** 


***Treatment of Bacillus Pyocyaneus Infection.*** By Kenneth Taylor, Director of the Laboratories, Robert Walton Goelet Research Fund. *Journal of the American Medical Association*, Nov. 25, 1916.

During two years of critical study of wound infection, the author found that the presence of Bacillus pyocyaneous, although never acutely dangerous, was the cause of delay in the repair of wounds. Most antiseptics are ineffective against it. It is rarely found in fresh wounds, but its incidence has increased after the first week, and it often remains the dominating infection.

It was found that B. pyocyaneus was rarely present when wound discharges were strongly acid, but was common when there was small acidity and especially when the reaction was neutral or alkaline. Therefore a number of acid antiseptics organic and inorganic were tested. The author notes that since the pigment pyocyanin is decolorized by most acids, the absence of the green color cannot be taken to indicate the absence of the B. pyocyaneus. Cultural tests must be used to determine the presence and vitality of the organism. Nitric and hydrochloric acid produced little effect upon the growth of the organism. Butyric, propionic, mallic, tartaric, and acetic acids were found to be much more effective. Acetic acid was much the most successful. .09 % concentration of this acid was required to inhibit growth in vitro against .94 % quinine hydrochlorid, 8. % sodium chlorid, and 36,420 % Dakin's solution. Sodium bicarbonate was found to stimulate the growth of the organism. A satured solution of benzoic acid was insufficient to inhibit growth. Cresol, however, gave even better results in vitro ( .08 %), but proved ineffective in clinical use.

In the wards of the hospital a series of 111 patients were dressed continuously by one of the solutions mentioned. To each fluid, except Dakin's, .8 % sodium chlorid was added. The incidence of B. pyocyaneus in wounds treated with acetic acid was 40 % on entry, 20 % after one week, and was completely inhibited thereafter. No other solution gave results at all comparable. With all the others except sodium chlorid, which stimulated growth and was discontinued, the organism increased up to the third week, and then rapidly declined. In cases treated with acetic acid, however, no organisms were found after the second week. Cresol showed a high incidence of B. pyocyaneous clinically and was therefore considered ineffective.

The author states that a 1 % solution of acetic acid has been used since these experiments as a routine procedure for the purpose in question, and has given satisfactory results.

